# Safety, Cognitive, and Behavioral Outcomes in Patients with Dementia with Lewy Bodies Treated with Nilotinib

**DOI:** 10.3390/jcm14124245

**Published:** 2025-06-14

**Authors:** Fernando Pagan, Yasar Torres-Yaghi, Michaeline Hebron, Barbara Wilmarth, R. Scott Turner, Sara Matar, Xiaoguang Liu, Dalila Ferrante, Giuseppe Esposito, Jaeil Ahn, Charbel Moussa

**Affiliations:** 1 Translational Neurotherapeutics Program, Laboratory for Dementia and Parkinsonism, Department of Neurology, Lewy Body Dementia Association Research Center of Excellence, Georgetown University Medical Center, Building D, Room 265, 4000 Reservoir Rd NW, Washington, DC 20057, USA; fernando.l.pagan@gunet.georgetown.edu (F.P.); yasar.a.torres-yaghi@gunet.georgetown.edu (Y.T.-Y.); mlh88@georgetown.edu (M.H.); barbara.m.wilmarth@gunet.georgetown.edu (B.W.); sm3469@georgetown.edu (S.M.); xl371@georgetown.edu (X.L.); df684@georgetown.edu (D.F.); 2 Department of Neurology, Movement Disorders Clinic, MedStar Georgetown University Hospital, PHC 7, 3300 Reservoir Rd NW, Washington, DC 20057, USA; 3 Memory Disorders Program, Department of Neurology, Georgetown University Medical Center, Suite 177, Building D, 4000 Reservoir Rd NW, Washington, DC 20057, USA; raymond.turner@georgetown.edu; 4 Division of Nuclear Medicine, Department of Radiology, MedStar Georgetown Hospital, Bles Building, 2nd Floor, 3300 Reservoir Rd NW, Washington, DC 20057, USA; exg11@gunet.georgetown.edu; 5 Department of Biostatistics, Bioinformatics and Biomathematics, Georgetown University Medical Center, Basic Science Building, 3800 Reservoir Rd NW, Washington, DC 20057, USA; ja1030@georgetown.edu

**Keywords:** Alzheimer’s disease, dementia with Lewy bodies, dopamine, falls, hyper-phosphorylated tau

## Abstract

**Background/Objectives:** We previously demonstrated that nilotinib can sufficiently enter the brain to pharmacologically inhibit discoidin domain receptors (DDR)-1 in patients with Parkinson’s and Alzheimer’s disease. We primarily hypothesized that nilotinib is safe, and may alter disease-related biomarkers to improve, motor, cognitive and/or behavioral features in dementia with Lewy bodies (DLB). **Methods**: Forty-three participants were randomized 1:1 into nilotinib, 200 mg, or matching placebo in a single-center, phase 2, randomized, double-blind study. Study drug was taken orally once daily for 6 months followed by one-month wash-out. **Results**: Of 43 individuals enrolled, 14 were women (33%); age (mean ± SD) was 73 ± 8.5 years. Nilotinib was safe and well-tolerated, and more adverse events were noted in the placebo (74) vs. nilotinib (37) groups (*p* = 0.054). The number of falls were reduced in the nilotinib (six) compared to placebo (21) group (*p* = 0.006). Cerebrospinal fluid homovanillic acid, a biomarker of dopamine levels, was increased (*p* = 0.004), while the ratio of pTau181/Aβ42 was reduced (*p* = 0.034). The Alzheimer’s Disease Assessment Scale—cognition 14 improved by 2.8 pts (*p* = 0.037), and no differences were observed in Movement Disorders Society–Unified Parkinson’s Disease Rating Scale parts II and III. However, part I (cognition) improved (*p* = 0.044) in nilotinib compared to placebo. **Conclusions**: Nilotinib demonstrates favorable safety, biomarkers, and efficacy outcomes in patients with DLB supporting further trials in DLB or advanced Parkinson’s disease with dementia.

## 1. Introduction

Dementia with Lewy Bodies (DLB) is the second most common form of dementia [1,2]. There is no cure or disease-modifying therapy for DLB, and symptomatic treatments provide limited benefits. Having effective, safe therapeutics to improve symptoms, function, and quality of life and to slow or halt progression of DLB is imperative. Dopaminergic (DA) replacement therapies may abate parkinsonian motor symptoms, but the risks of worsened psychosis and hypotension limit their use in many DLB patients. Selective Serotonin Re-uptake Inhibitors (SSRIs) and antipsychotics may help behavioral symptoms but can worsen motor features in DLB. Acetylcholinesterase inhibitors (AChEIs) may partially lessen cognitive symptoms in DLB but may also aggravate tremors or autonomic symptoms in some individuals.

No therapeutic approach exists to alter the levels of neurotoxic proteins and halt DA and other neuronal loss in DLB. Nilotinib inhibits tyrosine kinase Abelson (Abl, IC_50_ > 20 nM) [3] and is FDA-approved for Chronic Myelogenous Leukemia (CML) at oral doses of 300 mg twice daily. However, nilotinib more potently inhibits discoidin domain receptor (DDR)-1 (IC_50_ = 1 nM) [3]. Oral nilotinib, 150 mg and 300 mg, once daily demonstrate a linearly proportional increase to dose in the brain of Alzheimer’s disease (AD) [4] and Parkinson’s disease (PD) [5] patients and reach a CNS concentration (1–5 nM) that directly inhibits DDR1 (IC_50_ 1 nM) [6], thus exhibiting an adequate pharmacokinetics and pharmacodynamics relationship. A dose-dependent increase in CNS DA metabolite, including homovanillic acid (HVA), was detected when individuals with PD or AD were treated with nilotinib, 150 mg or 300 mg.

An open-label study suggested that nilotinib may be beneficial in advanced PD with dementia (PDD) and DLB [7], but subsequent two independent phase 2 studies in moderately advanced PD (stage 2.5–3) showed that nilotinib did not have a symptomatic motor effect in medically optimized patients at 6 months [5,8] and 12 months [5]. Long-term treatment (27 months) suggested that nilotinib 300 mg, significantly stabilized motor and cognitive symptoms in PD patients with mild cognitive impairment (MCI) [9].

Here we report the findings of a phase 2, single-center, double-blind, placebo-controlled study using a new dose of nilotinib (200 mg) to primarily evaluate the safety and tolerability of this dose in DLB. Our secondary aims include disease-related biomarkers, including CSF DA, alpha-synuclein, phospho-tau181 (ptau) and amyloid and clinical outcomes.

## 2. Materials and Methods

### 2.1. Standard Protocol Approvals and Registrations

This is a single-site study that was conducted by the Translational Neurotherapeutics Program (TNP) at Georgetown University Medical Center (GUMC). This study was approved by the Institutional Review Board (STUDY00000122, August 2019) at GUMC. The study was conducted under FDA Investigational New Drug (IND) # 142271 and clinical trial number NCT04002674 in August 2019.

### 2.2. Randomization and Blinding

This study employed block randomization using block and function in R software (ver. 3.4) to randomize 60 participants into the 2 treatment groups. The block size varies between baseline and 26 weeks, and the randomization was performed within blocks to ensure a balance in sample sizes across groups blocks. Site staff, investigators, raters, patients and study partners were blinded to treatment.

### 2.3. Participants

Participants and caregivers consented in writing and complied with study procedures according to protocol. Inclusion criteria included participants who are medically stable, males and females (25–90 years), multi-racial groups with clinical diagnosis of probable DLB [10], including dementia with Montreal Cognitive Assessment (MoCA) ≥ 18 and Parkinsonism. Participants Unified Parkinson’s Disease Rating Scale (UPDRS) I-III was less than 50 and UPDRS-III between 20 and 40. An abnormal (historical) DAT scan was provided to lend further support to diagnosis. Participants were stable on Levodopa but monoamine oxidase (MAO_ inhibitors, e.g., Rasagiline were excluded to prevent changes in brain HVA levels. Participants with QTc interval 350–470 ms were enrolled with stable concomitant medical and/or psychiatric illnesses. Consent was provided for lumbar punctures at baseline and 6 months. Conditions that did not conform to LBD diagnosis, other forms of dementia or movement disorders, any unstable physical or psychiatric symptoms were excluded from the study.

### 2.4. Study Design and Objectives

The aim of this study was to evaluate the safety and tolerability of 200 mg nilotinib in individuals with DLB. The primary objective included nilotinib pharmacokinetics. Secondary objectives included biomarkers, primarily DA metabolism, and exploratory clinical outcomes. Participants (*n* = 43) were randomized (1:1) into oral 200 mg nilotinib (*n* = 21) or placebo (*n* = 22) daily for 6 months followed by 1-month washout. Lumbar puncture (LP) was performed after dosing. Safety was measured using Adverse Events (AEs) and serious AEs (SAEs) deemed to be possibly related, probably related, or related to the study drug.

### 2.5. Sex as a Biological Variable

Men and women were included in this study as sex may be a biological variable. However, due to the small number of subjects, stratification by sex or gender was not performed.

### 2.6. Data Analysis

Baseline characteristics were descriptively summarized using mean and standard deviation (SD) for continuous variables such as age and drug dose and frequencies and percentages for categorical variables by the two treatment groups. For comparisons between the two groups, two sample t-tests for continuous variables or Pearson’s chi-squared test for the binary variables was used. SAEs and AEs were summarized using frequencies and percentages by the two treatment groups and the SAE counts were compared using the exact Poisson method. Secondary biomarkers are presented as mean  ±  SD in charts. The changes in biomarkers within each group were compared using *t*-tests. Exploratory clinical endpoints by the groups at baseline, 12 weeks, and 26 weeks were summarized using sample mean ± SDs along with trajectory plots of changes over visits. To account for discrete score changes with massive ties, Wilcoxon signed-rank tests and their 95% confidence intervals (CI) were used to test whether there are treatment group differences in the changes in each clinical endpoint between baseline and 26 weeks, respectively. Statistical significance was determined if a two-sided *p* < 0.05. Analyses of secondary and exploratory endpoints were designed for proof-of-concept and no multiplicity correction was applied. In this proof-of-concept study, 43 patients were acceptable to provide first evidence of efficacy by offering effect sizes on efficacy and biomarker outcomes and by providing detailed descriptive analyses of changes in outcomes in each period. Due to the premature end of trial, the statistical power has decreased, and the minimal detectable power was 0.9 SD at 80% power at a two-sided alpha of 5%. Statistical analyses for demographic and clinical parameters were conducted using Rstudio, ver. 1.5, and biomarker analysis was performed using GraphPad Prism, version 9.1.2 (GraphPad software Inc., Boston, MA, USA).

### 2.7. Plasma and CSF Collection

Blood draw (10 mL) and LP ~15 mL CSF were performed at baseline and end of treatment (EOT) at six months, as we previously described [5].

### 2.8. Total Alpha-Synuclein ELISA

We used solid phase sandwich ELISA to quantify CSF alpha-synuclein according to manufacturer’s protocol (Cat#844101, Biolegend, San Diego, CA, USA) as we previously described [5].

### 2.9. Aβ40, Aβ42, Total Tau, and p-Tau 181 ELISA

CSF samples were analyzed in parallel using the same reagents, using Milliplex ELISA (Cat. #HNABTMAG-68 K, Millipore, Danvers, MA, USA) according to protocol as we previously described [4].

### 2.10. Quantification of Dopamine Metabolite HVA

The concentrations of HVA in the CSF samples were measured by UHPLC-MS/MS following derivatization with benzoyl chloride, using Pronexus Analytical AB, Bromma, Sweden as we previously described [5]. Nilotinib, as mentioned in the protocol, was taken on an empty stomach for at least 2 h. Blood and CSF collections were performed half an hour apart, and were conducted 1, 2, 3, and 4 h after nilotinib administration. Therefore, the 4 h time point was the duration of fasting during this study.

### 2.11. Clinical Assessments

All participants were tested less than 2 h since the last dose of levodopa. A single rater conducted all clinical exams in all participants across all study visits. All clinical assessments were performed at baseline, 12 weeks, and 26 weeks via MoCA [11], MDS-UPDRS [12], Alzheimer’s Disease Assessment Scale—cognition 14 (ADAS-cog14) [13], Alzheimer’s disease Cooperative Study—Activities of Daily Living (ADCS-ADL) [14], clinical assessment of fluctuation (CAF) [15], irritability assessment scale (IAS) [16], Problem Behavior Assessment (PBA) [17], and Neuropsychiatric Inventory (NPI) [18].

### 2.12. Positron Emission Tomography

Florbetaben F-18 Positron Emission Tomography (PET) imaging was used to estimate β-amyloid (Aβ42) neuritic plaque density. SUVr (SUV ratio) data were obtained for each anatomical region by dividing the average regional counts by the average counts of the cerebellar cortex as we previously described [4]. Z-scores are produced for each region, from the statistical comparison of regional SUVr to the reference population. Region based statistical analysis of the amyloid PET images was conducted using Brass Software (https://www.hermesmedical.com/, accessed on 5 June 2025, Hermes Medical Solutions, Stockholm, Sweeden). Each PET image was stereotactically normalized to a reference template created from images of normal subjects.

## 3. Results

### 3.1. Enrollment and Demographics

**Figure 1:**
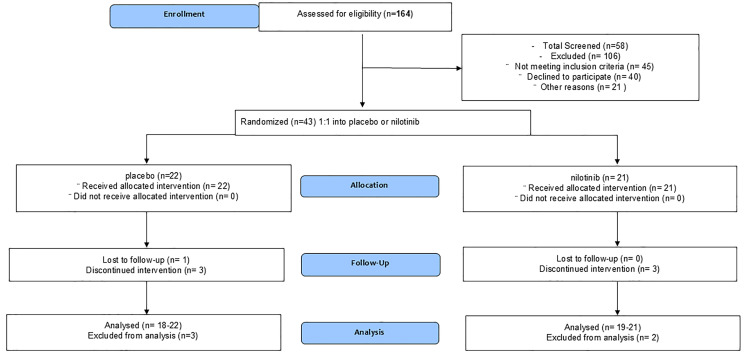
Consolidated standards of reporting trials (CONSORT). Phase 2, randomized, double-blind, placebo-controlled study to evaluate nilotinib effects on safety, tolerability, and clinical outcomes in dementia with Lewy bodies.

Of 164 subjects approached, 58 were screened, 45 did not meet inclusion/exclusion criteria, 43 were randomized, and 7 self-withdrew during the screening period (Figure 1—CONSORT and Table 1).

Participants included male and female (14:7) in the nilotinib and (15:7) in the placebo arm with an average age of 73 ± 8.5 (year ± SD). Thirty-six (36) participants, including eighteen (81%) in placebo and eighteen (86%) in nilotinib, completed all study procedures as per protocol, and four (18%) participants withdrew from placebo (one during washout) and three (14%) participants withdrew from nilotinib. The placebo group included 18 non-Hispanic whites, one black, one Asian, and two whites with no reported ethnicity. The nilotinib group included sixteen non-Hispanic whites, one Asian, and four whites with no reported ethnicity.

Dementia and cognitive fluctuations were chief medical complaints in all participants. Parkinsonism was another symptomatic feature, and most patients exhibited recurrent falls and imbalances without other parkinsonian symptoms. A total of six patients exhibited psychiatric symptoms, including hallucinations and were stabilized on daily doses of pimavanserin and quetiapine. MoCA score was 19 ± 3.3 (mean ± SD) in the nilotinib and 19 ± 5.2 (mean ± SD) in the placebo group. MDS-UPDRS-Part III (motor) was 19 ± 7.3 (mean ± SD) in nilotinib and 19 ± 9.19 (mean ± SD) in placebo. Average Levodopa Equivalent Daily Dose (LEDD) was 164 ± 227 mg at enrollment and 160± 258.5 mg (mean ± SD) at six months in the nilotinib group, suggesting a 5.5% decrease. LEDD was 126 ± 200 mg at enrollment and 151 ± 254 mg (mean ± SD) at six months in the placebo group, suggesting a 37% increase. Levodopa included Rytary and Sinemet, and there were no MAO-B inhibitors (excluded) or DA agonists. AChEIs, including therapeutic doses of transdermal rivastigamine (Exelon^®^ patch), Namzaric, Namenda, and donepezil were not changed in both groups. Four participants were on antipsychotics at inclusion, including therapeutic doses of quetiapine and pimavanserin in the placebo group and one participant was on Pimavanserin in the nilotinib group. There was no change in antidepressants and anxiolytics from baseline to month six among all participants.

### 3.2. Adverse Events

There were two SAEs in the placebo group (Table 1), including atrial fibrillation in one participant and an appendectomy in another. There were two SAEs, including one incident of worsening dyskinesia from baseline and one fall that led to hospitalization in the nilotinib group. There were 37 AEs (Table 2) in the nilotinib group and 74 in the placebo group (incidence ratio 95% CI, 0.98–2.32, *p* = 0.054). The most common AE was fall in the placebo group (21), and this was significantly reduced in the nilotinib (six) group (95% CI, 1.30 to 10.12, *p* = 0.006). Common AEs included coronavirus (COVID-19) infection in the placebo (81%) and nilotinib (14.3%) groups and joint and muscle pain in the placebo (31.8%) and nilotinib (19%) groups. Hallucinations were reported in placebo (13.8%), and skin lesions were reported in the nilotinib (19%) group. All other AEs were less common and rare (<10%) (Table 2).

### 3.3. Biomarkers

**Figure 2:**
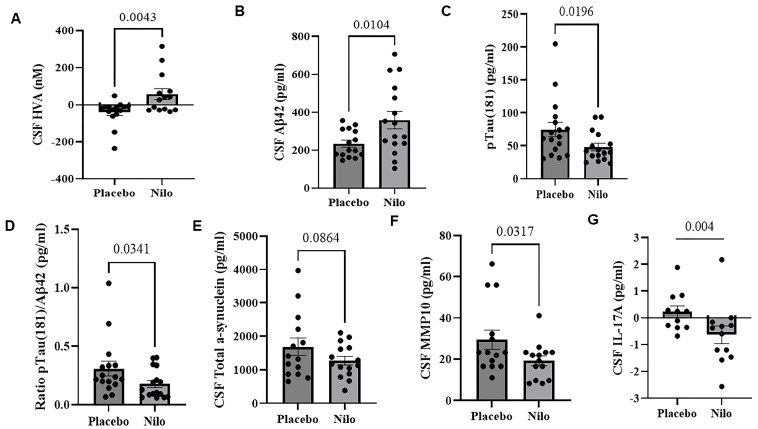
The effects of nilotinib on biomarkers. Histograms represent cerebrospinal fluid (CSF) levels of the mean difference between nilotinib and placebo groups of (**A**) homovanillic acid (HVA), (**B**) beta amyloid (Aβ42), (**C**) hyperphosphorylated tau at serine 181 (ptau 181), (**D**) ratio of ptau181/Aβ42, (**E**) alpha-synuclein, (**F**) matrix metalloproteases (MMP)10, and (**G**) interleukin (IL)-17A.

There was a significant decrease in CSF DA metabolite HVA levels between baseline and end of treatment (EOT) values. The mean average difference in CSF HVA was significantly increased (Figure 2A, Supplementary Table S1, 98.53 ± 34.06 nM, 95% CI, 27.81 to 169.3, *p* = 0.0043) in the nilotinib compared to the placebo group. The level of CSF Aβ42 was increased (Figure 2A, Supplementary Table S1, 124.5 ± 49.60 nM, 95% CI, 20.96 to 228.0, *p* = 0.0104), while p-Tau181 was reduced (Figure 2C, Supplementary Table S1 −26.75 ± 12.26 nM, 95% CI, −52.06 to −1.441, *p* = 0.0196) in the nilotinib group compared to the placebo. The CSF ratio of pTau181/Aβ42 was reduced (Figure 2D, Supplementary Table S1, −0.13 ± 0.068 nM, 95% CI, −0.2 to 0.01, *p* = 0.0341), and alpha-synuclein trended towards a decrease (Figure 2E, Supplementary Table S1, −416.5 ± 293.9 nM, 95% CI, −1032 to 198.8, *p* = 0.086) in nilotinib versus placebo.

CSF levels of matrix metalloproteases (MMP)-10 were reduced in nilotinib (Figure 2F, Supplementary Table S1, −10.21 ± 5.185 nM, 95% CI, −21.04 to 0.63, *p* = 0.0317) and pro-inflammatory marker interleukin (IL)-17a (Figure 2G, Supplementary Table S1) also decreased (−0.94 nM, CI 95%, −1.51 to −0.38, *p* = 0.004). On average, there was a trend toward a decrease in Aβ42 burden via PET on SUVr at six months in nilotinib group in frontal (−0.13) and temporal lobes (−0.09) compared to placebo (0.04 and 0.03, respectively), albeit these changes were not significant (Supplementary Tables S2 and S3). No significant differences in amyloid PET were observed at baseline (Supplementary Table S4). It is also worth mentioning that the ratio of Aβ42/Aβ40 as an important diagnostic and prognostic biomarker of diseases (Supplementary Table S1) is not significant, and the level of Aβ40 is reduced in both the placebo and nilotinib groups over 6 months, and there were no statistical differences between the groups. The ratio of Aβ42/Aβ40 may correlate with the PET data due to different diagnosis of DLB compared to AD.

### 3.4. Clinical Outcomes

**Figure 3:**
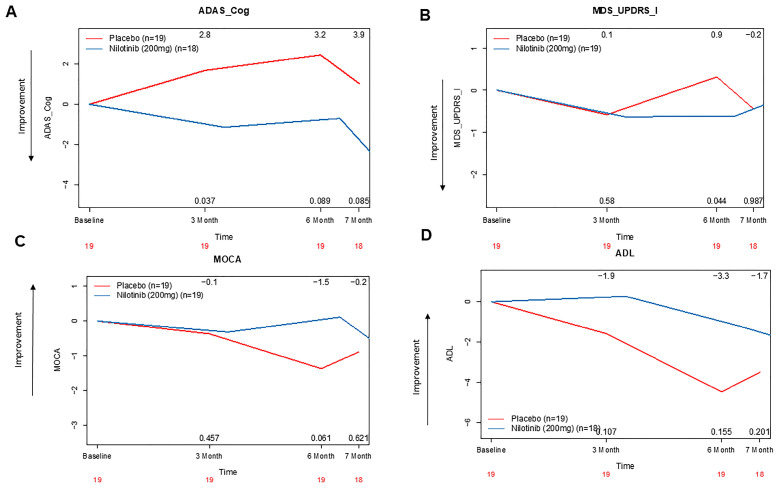
The effects of nilotinib on cognitive outcomes over time. Graphs represent changes in scores on (**A**). Alzheimer’s Disease Assessment Scale—cognition 14 (ADAS-cog14), (**B**). Movement Disorders Society-Unified Parkinson’s Disease Rating Scale (MDS-UPDRS)-part I (cognition), (**C**). Montreal Cognitive Assessment (MOCA) and (**D**). Alzheimer’s Disease Cooperative Study—Activities of Daily Living (ADL). All values are anchored on baseline.

An improvement in ADAS-Cog14 (memory, language and praxis) score was observed at three months (2.8 pts, 95% CI, 95% CI, 0 to 6.34, *p* = 0.037) and six months (3.2 pts, 95% CI, −0.67 to 7.34, *p* = 0.08) in the nilotinib versus placebo group (Figure 3A, Supplementary Tables S5 and S6).

No differences were observed in MDS-UPDRS Part II (activities of daily living) and Part III-motor (Supplementary Tables S5 and S6). MDS-UPDRS—Part I (cognition) improved (Figure 3B) at six months (0.9 pts, 95% CI, 0 to 2, *p* = 0.044) and this improvement in cognition was supported by a trend in MoCA scores (1.5 pts, 95% CI, 0 to 3, *p* = 0.061) between the nilotinib and placebo groups at six months (Figure 2C). The mean difference in ADCS-ADL scores improved at six months (Figure 2D) in nilotinib compared to placebo (−3.3 pts, 95% CI, −5 to 1, *p* = 0.084).

**Figure 4:**
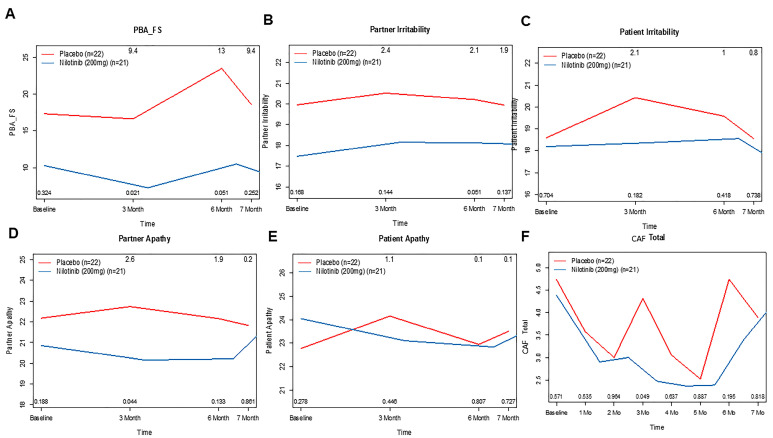
The effects of nilotinib on behavioral outcomes over time. Graphs represent changes in scores on (**A**) frequency and severity of problem behavior assessment—small version (PBAs), (**B**) irritability reported by study partner, and (**C**) patient measured via irritability assessment scale (IAS). Apathy reported by (**D**) study partner and (**E**) patient. (**F**) Clinical assessment of fluctuation (CAF) from month to month in placebo compared to nilotinib group.

Behavioral and psychiatric manifestations (e.g., depression, suicide, anxiety, irritability, anger, perseveration, OCD, apathy, delusions, and hallucinations) were assessed via the short version of PBA-s caregiver interviews, showing that the frequency and severity (Figure 4A) of these symptoms were higher in the placebo group at three months (9.4 pts, 95%, CI, 1 to 13, *p* = 0.021) and six months (13 pts, 95%, CI, 0 to 16, *p* = 0.051) compared to nilotinib. These features were also evident in the study partner’s account of irritability (Figure 4B) at three months (2.1 pts, 95%, CI, −1 to 4, *p* = 0.051) as well as participants’ reports of irritability (Figure 4C). Study partners report of apathy (Figure 4C) at 6 months (2.6 pts, 95%, CI, 0 to 4, *p* = 0.044) and patient report of apathy (Figure 4E) were not significant. CAF was scored upon the clinician’s interpretation of partner responses to fluctuating confusion and impaired consciousness during the month prior to the assessment (Figure 4F) and demonstrated a steady decrease (50%) in score from month to month (4.5 pts at baseline–2.2 pts at month six) and was significant at three months (*p* = 0.049), compared to the placebo group.

## 4. Discussion

Thirty-six (36) participants (83.7%) completed all study procedures, which anticipated 60 participants to be enrolled, falling behind the targeted enrollment (71.7%) due to the COVID-19 pandemic, which resulted in major disruptions in recruitment. Participants were both male (29) and female (14), with an average age of 73 ± 8.5 (year ± SD). No dropouts or SAEs due to adverse drug effects were reported, indicating that nilotinib was well-tolerated. There was an increase in LEDD from baseline to EOT in the placebo (37%) compared to the nilotinib group over six months, but the two groups averaged equally around 19 pts on MDS-UPDRS-Part III (motor), indicating mild motor symptoms. AChEIs were not changed in both groups, and MoCA averaged around 19 pts, indicating moderate dementia in all participants. Antipsychotics were stable, although the placebo group reported more hallucinations than nilotinib.

Seven participants withdrew from the study, including four from the placebo group. One patient was withdrawn by the PI (at month three) due to a history of hallucinations that was exacerbated from baseline in addition to non-compliance with study requirements, including initiating AChEIs mid-study. Three other patients self-withdrew—one due to appendectomy, and one due to COVID-19 at month two, while another completed all procedures for six months (EOT) and did not show up to the follow-up visit. In the nilotinib group, one patient self-withdrew due to COVID-19; another was hospitalized due to fall and worsening orthostatic hypotension and withdrew two months after baseline. One patient had worsening dyskinesia which occurred five days after starting study drug and was hospitalized and discontinued the study. This study also showed fewer AEs (37) in the nilotinib compared to the placebo group (74), and hallucinations were reported in placebo, while skin lesions were reported in the nilotinib group. All other AEs were less common and rare (<10%), indicating nilotinib safety (200 mg daily). Nilotinib is FDA approved for CML, and several clinical trials and the prescriptions information of oral nilotinib (>600 mg daily) include warnings and precautions, including QTc prolongation, myelosuppression, elevation of liver transaminases, renal toxicity, and fluid retention. All participants were monitored via blood tests and complete blood count (CBC) according to protocol, and no side effects were observed throughout the study. This study used a lower dose of nilotinib, 200 mg, in addition to two previous studies (*n* = 150 patients) in PD [5,8] and one study (*n* = 43 patients) in AD [4] using nilotinib, 150 mg and 300 mg. Therefore, a dose range of nilotinib of 150–300 mg demonstrates an acceptable safety and tolerability profile compared to 600 mg in CML.

The significantly lower number of falls in the nilotinib group compared to placebo, which was also concurrent with improved cognition, suggests that better cognition may increase stability and reduce falls in patients with DLB. These results are consistent with the literature, suggesting that non-AD dementia is associated with increased falls compared to cognitively healthy individuals [19]. Nilotinib treatment appears to meaningfully improve cognition and reduce behavioral symptoms and cognitive fluctuations as early as three months. Although no motor differences were noted on MDS-UPDRS Part III or total (Part I + II + III), significant differences were observed on cognition (UPDRS-Part I), further suggesting that nilotinib improves cognitive outcomes in moderately demented DLB patients. The dilemma of currently approved-FDA symptomatic drugs that improve motor but worsen cognitive and/or behavioral symptoms, or vice versa, is a significant medical issue, and this study suggests that nilotinib 200 mg, may overcome this conundrum. A seminal 2016 report of an open-label study (*n* = 11 patients) suggested that nilotinib may be beneficial in advanced PD with dementia (PDD) and DLB patients [7]. Two independent placebo-controlled, phase 2a studies of nilotinib in moderately advanced PD-MCI [5] and mild-moderate PD [8] showed that nilotinib did not have a symptomatic motor effect in medically optimized PD patients at six months [5,8] and twelve months [5]. However, long-term treatment (27 months) with nilotinib, 300 mg, stabilized motor and cognitive outcomes and improved PD Quality of Life (PDQ)-39 in PD-MCI [9]. Taken together, these findings suggest that patients with Lewy Body Dementia (LBD)—the umbrella term of DLB and PDD—may experience an improvement in quality of life due to cognitive and/or behavioral improvement, and the stabilization of motor symptoms.

Biomarker studies supported nilotinib effects of cognition as the level of DA metabolite HVA was increased. DLB is characterized by the death of nigrostriatal DA neurons; however, DA can influence executive functions via DA neurons in the mesolimbic system, and this role may be particularly relevant to cognition in DLB [20,21]. A dose-dependent increase in CNS DA was detected when individuals with PD-MCI or levodopa-naïve AD patients were treated with nilotinib, 150 mg and 300 mg [4,5]. The effects of nilotinib on CNS DA is associated with decreased catabolism via monoamine oxidase-A (MAO-A) inhibition [9] and downregulation of catechol-O-methyltransferase (COMT) genes [6]. These data are also consistent with pre-clinical evidence of nilotinib reversing DA neuron dysfunction in the ventral tegmental area (VTA) and mesocorticolimbic system, leading to cognitive improvement [22]. An increase in brain DA may cause cognitive improvement in moderately demented DLB patients, independent of mild motor symptoms. The level of HVA was reduced in the CSF between baseline and EOT, despite the increase in LEDD in the placebo group, suggesting loss of DA. Conversely, LEDD was stable between baseline and EOT, but HVA was increased in the nilotinib group, suggesting an increase in brain DA.

DLB is an alpha-synucleinopathy that is characterized by formation of Lewy Body (LB) inclusions consisting of aggregated alpha-synuclein. Hyper-phosphorylated tau (p-tau) and Aβ peptides are also present throughout the brain in LBD. Our data showed that nilotinib reduced the level of CSF p-Tau 181. CSF Aβ42 increased, suggesting less CNS amyloid plaque deposition. However, the lack of statistical significance in changes in Aβ plaques via PET between nilotinib and placebo in this study may be due to the short term of the 6-month treatment with 200 mg nilotinib compared to previous 1-year treatment with up to 300 mg nilotinib in AD patients [4]. The observed ratio of Aβ42 /Aβ40 is an important diagnostic and prognostic biomarker of AD and it correlates with the PET results in this study, suggesting that a different diagnosis of DLB compared to AD may be associated with different ratio of Aβ42/Aβ40 or plaque depositions in the brain. Additionally, the small sample size, 6-months (short) duration of treatment or a potentially mixed pathology (alpha-synuclein, p-tau and Aβ40 and Aβ42 observed in DLB [23,24,25,26] may prevent observation, or lack, of statistical differences between groups. We reported a reduction in genes via miRNAs that control production of chemokines and cytokines and other inflammatory markers in the CSF of nilotinib-treated AD patients compared to placebo [6], in agreement with CSF reduction in IL-17a and pre-clinical data that DDR1 inhibition facilitates autophagy and reduces inflammation [27]. Caspase-3 gene expression—a biomarker of apoptotic cell death—was reduced with nilotinib treatment [6], consistent with volumetric magnetic resonance imaging (vMRI), showing reduced hippocampal atrophy in nilotinib-treated AD patients [4].

FDA approved treatments for early AD such as lecanemab [28] and donanemab [29] showed a significant increase in amyloid related imaging abnormalities (ARIA) due to brain bleeds, while next generation whole genome sequencing of CSF miRNAs from nilotinib treated mild-moderate AD [6] and PD-MCI [30] patients showed favorable changes in genes that regulate vascular fibrosis, including the master regulator of fibrosis Transforming Growth Factors (TGFs) [31] and Tissue Inhibitors of Metalloproteases (TIMPs) that control the expression and function of MMP [32]. Nilotinib reduces DDR-1 expression [6] and reduces its phosphorylation (activation) in the CSF of AD patients [6], while the activation of DDR1 increases microglial activity and MMPs [33,34]. Our data show a decrease in MMP10 in nilotinib versus placebo groups, indicating that nilotinib may affect fibrosis and inflammation in DLB. CSF miRNA sequencing from PD-MCI treated with 150 mg or 300 mg nilotinib for twelve months revealed involvement of molecular pathways that regulate angiogenesis and the blood–brain barrier, neuro-inflammation, and autophagy. Taken together, these findings suggest that nilotinib attenuates inflammation and may suppress fibrosis in agreement with animal models [35,36], and these findings suggest that nilotinib may be protective against ARIA observed with antibody treatments [28,29].

Nilotinib is available for prescription use and demonstrated an acceptable safety profile in humans, and our data are encouraging and provide solid grounds to investigate nilotinib effects on cognition. In AD, nilotinib 150 mg may improve executive functions, but the higher 300 mg dose increased (70%) agitation and irritability [4], suggesting that a lower dosage is more adequate [4], hence the selection of 200 mg dosage. The occurrence of behavioral side effects with nilotinib 300 mg may be due to the increase in CNS DA, and our current study demonstrates that nilotinib 200 mg increases CNS DA without behavioral and/or motor side effects. Therefore, nilotinib is an oral, low-dose (200 mg) therapy that may prevent the accumulation of amyloid and tau, protect blood vessel fibrosis, and prevent inflammation in the spectrum of dementia and cognitive decline.

A potential shortcoming of this study is the small sample size from a single center. Although ADAS-cog14 showed nilotinib effects on cognition in moderately demented patients, the inclusion of more patients across the dementia continuum due to early AD, PDD, and DLB is preferable to study nilotinib in larger, multicenter studies, with longer treatment periods (>6 months). Future studies should also measure seeding of alpha-synuclein in CSF to evaluate whether nilotinib can affect misfolding of alpha-synuclein.

## 5. Conclusions

The FDA-approved drug nilotinib is safe and provides a strategic option that may lead to a repurposed drug in LBD (DLB and PDD) that is now generic in the US and can help improve cognition and reduce behavioral symptoms and falls, thus attenuating the burden for patients and caregivers. The favorable effects of nilotinib (200 mg) on cognition, without worsening motor and/or behavioral symptoms, may help overcome the dilemma of currently approved symptomatic drugs that improve motor but worsen cognitive symptoms, or vice versa.

## 6. Patents

Charbel Moussa is an inventor at Georgetown University (GU) US and holds an international patent for the use of niloitnib in neurodegenerative diseases, including alpha-synucleinopathies. GU has exclusively licensed the nilotinib patent to KeifeRx LLC. Charbel Moussa and Fernando Pagan are co-founders and shareholders of KeifeRx LLC, and Charbel Moussa is a paid consultant for KeifeRx LLC. All other authors declare no conflicts of interests regarding this manuscript.

## Figures and Tables

**Table 1:** Demographics and summary of all reported serious adverse events in nilotinib and placebo groups. SD: standard deviation.

Demographics	Placebo	Nilotinib (200 mg)
Total enrolled	*n* = 22	*n* = 21
Total finished end of Treatment	18 (81%)	18 (86%)
Total dropped out	4 (18%)	3 (14%)
Average age (years) ± SD	73 ± 7	73 ± 10
Weight (kg) ± SD	74 ± 15	74 ± 15
Height (cm) ± SD	172 ± 9	172 ± 9
Body Mass Index (BMI) ± SD	25 ± 3	25 ± 3
Male	15 (68%)	14 (66.6%)
Female	7 (32%)	7 (33.3%)
Race	18 whites (81.8%) non-Hispanic	16 whites (76.2%) non-Hispanic
	1 Black (4.5%)	0 (0%)
	1 Asian (4.5%)	1 Asian (4.8%)
	2 whites (9.2%) not reported	4 whites (19%) not reported
Montreal Cognitive Assessment (MoCA) at screening Mean ± SD	19 ± 5.2	19 ± 3.3
Unified Parkinson’s Disease Rating Scale (UPDRS)-Part 3	19 ± 9.19	19 ± 7.30
Levodopa Equivalent Daily Dose (LEDD) at baseline	126 mg ± 200	164 mg ± 227 (37%)
Levodopa Equivalent Daily Dose (LEDD) at 26 weeks (% increase)	151 mg ± 254	160 mg ± 258.5 (5.5%)
Acetylcholinesterase inhibitors at baseline	11.7 mg ± 4.75	11.7 mg ± 4.75 (0%)
Acetylcholinesterase inhibitors at 26 weeks (% increase)	10.6 mg ± 3.4	10.6 mg ± 3.4 (0%)
Antidepressants and Anxiolytics	No change	No change
Antipsychotics	No change	No change
Serious Adverse Events (SAEs)		
Event	Number of events (%)	Number of events (%)
Atrial fibrillation	1 (4.5%)	0 (0%)
Appendectomy	1 (4.5%)	0 (0%)
Dyskinesia	0 (0%)	1 (4.76%) (baseline)
Fall	0 (0%)	1 (4.76%) (baseline)
Total SAEs	2	2

**Table 2:** Summary of all reported adverse events in nilotinib and placebo groups. NOSD: not on study drug.

Adverse Events (AEs)			
System Organ Class		Placebo (*n* = 22)	Nilotinib (*n* = 21)
	Preferred Term	Number of events (%)	Number of events (%)
Cardiovascular Disorders	Atrial fibrillation (NOSD)	1 (4.5%)	
	Ecchymosis	2 (9%)	
Gastrointestinal Disorders	Diarrhea	1 (4.5%)	
	Nausea		1 (4.76%)
	Constipation		1 (4.76%)
	Stomach bug		1 (4.76%)
General Disorders	Falls	21 (95.5%)	6 (28.6%)
	Shingles	1 (4.5%)	1 (4.76%)
	COVID-19	18 (81%)	3 (14.3%)
	Heavy chest	1 (4.5%)	
	Ear infection	1 (4.5%)	
	Sore throat		1 (4.76%)
	Low serum phosphorous (NOSD)		1 (4.76%)
	Joint and Muscle pain	7 (31.8%)	4 (19%)
	Rotator Cuff Tear		1 (4.76%)
Eye Disorders	Cataract	1 (4.5%)	
Nervous System Disorders	Post-Lumbar Puncture Headache		1 (4.76%)
	Wandering at night	1 (4.5%)	
	Agitation	1 (4.5%)	
	Hallucinations	3 (13.6%)	
	Delirium (COVID)		1 (4.76%)
	Tremor/akinesia		1 (4.76%)
	Blood patch		1 (4.76%)
Renal and Urinary Disorders	Kidney stone	1 (4.5%)	1 (4.76%)
	Urinary Tract Infection	3 (4.5%)	1 (4.76%)
	Hematuria		1 (4.76%)
	Cystoscopy	1 (4.5%)	
	Prostate (PSA elevation)	1 (4.5%)	
Respiratory, Thoracic, and Mediastinal Disorders	Upper Respiratory infection	2 (9%)	1 (4.76%)
Skin and Subcutaneous Disorder	Abscess	1 (4.5%)	
	Gout	1 (4.5%)	
	Lesions	1 (4.5%)	4 (19%)
	Tissue mass	2 (9%)	
	Rash	2 (9%)	4 (19%)
	Mohs Procedure		1 (4.76%)
Total AEs		74	37

## Data Availability

The final data, study protocol, and all interpretations will be available to the scientific and non-scientific community and clinicians upon request (to CM). Investigators adhered to the Privacy Rule under the Health Insurance Portability and Accountability Act (HIPAA).

## Supplementary Materials

The following supporting information can be downloaded at https://www.mdpi.com/article/10.3390/jcm14124245/s1. Table S1—Summary of mean values at baseline and end of treatment (EOT) of analyzed biomarkers, including cerebrospinal fluid (CSF) values of total alpha-synuclein (α-syn), homovanillic acid (HVA), beta amyloid (Aβ40 and Aβ42), total tau (tTau) and phosphorylated at serine 181 (pTau181), matrix metalloproteases (MMP)-10 and interleukin (IL)17A. Wilcoxon one tailed t-test. Baseline and EOT MEAN ± SD have been adjusted in accordance with the statistical analysis. Table S2—Florbetaben F-18 Positron Emission Tomography (PET) imaging was used to estimate β-amyloid (Aβ42) changes from baseline to 6 months was quantified using SUVr in different brain regions, including cortex (Ctx), cingulate, lateral temporal, cerebellum, and pons. A decrease in Aβ42 burden was trending in frontal cortex and lateral temporal lobe. Table S3—Relative changes from Baseline to 6 months in Florbetaben F-18 Positron Emission Tomography (PET) imaging to estimate β-amyloid (Aβ42) changes in different brain regions, including cortex (Ctx), cingulate, lateral temporal, cerebellum, and pons. PET Scan Descriptive Statistics of SUVr. Table S4—Baseline Florbetaben F-18 Positron Emission Tomography (PET) assessments using descriptive Statistics of SUVr for 16 patients in each group showing variability of amyloid burden. No significant differences were observed in baseline PET between placebo and nilotinib group. Table S5—Descriptive statistics of clinical assessments at baseline via Montreal Cognitive Assessment (MoCA), movement disorders society—unified Parkinson’s disease scale (MDS-UPDRS) part I, II and III, Alzheimer’s disease assessment scale—cognition 14 (ADAS-cog), Alzheimer’s disease cooperative study- Activities of Daily Living (ADL), clinical assessment of fluctuation (CAF), irritability assessment scale (IAS), problem behavior assessment (PBA), neuropsychiatric inventory (NPI) and trail making test (TMT)-B. Table S6—Descriptive statistics of clinical assessments at baseline and 6 months via Montreal cognitive assessment (MoCA), movement disorders society- unified Parkinson’s disease scale (MDS-UPDRS) part I, II and III, Alzheimer’s disease assessment scale—cognition 14 (ADAS-cog), Alzheimer’s disease cooperative study- Activities of Daily Living (ADL), clinical assessment of fluctuation (CAF), irritability assessment scale (IAS), problem behavior assessment (PBA), neuropsychiatric inventory (NPI) and trail making test (TMT)-B. Table S7—Descriptive statistics of clinical assessments at baseline via Montreal Cognitive Assessment (MoCA), movement disorders society- unified Parkinson’s disease scale (MDS-UPDRS) part I, II and III, Alzheimer’s Disease assessment scale—cognition 14 (ADAS-cog), Alzheimer’s disease cooperative study—Activities of Daily Living (ADL), clinical assessment of fluctuation (CAF), irritability assessment scale (IAS), problem behavior assessment (PBA), neuropsychiatric inventory (NPI) and trail making test (TMT)-B. Table S8—Descriptive statistics of changes from baseline to 6 months of Montreal Cognitive Assessment (MoCA), movement disorders society-unified Parkinson’s disease scale (MDS-UPDRS) part I, II and III, Alzheimer’s disease assessment scale—cognition 14 (ADAS-cog), Alzheimer’s disease cooperative study—Activities of Daily Living (ADL), clinical assessment of fluctuation (CAF), irritability assessment scale (IAS), problem behavior assessment (PBA), neuropsychiatric inventory (NPI) and trail making test (TMT)-B.

## Abbreviations

The following abbreviations are used in this manuscript:

DLB	Dementia with Lewy bodies
AD	Alzheimer’s disease
LP	Lumbar puncture
PD	Parkinson’s disease
Aβ	Amyloid-beta
LEDD	Levodopa equivalent daily dose
EOT	End of treatment
MCI	Mild cognitive impairment
MoCA	Montreal cognitive assessment
CAF	Clinicians assessment of fluctuation
ADAS-Cog	Alzheimer’s disease assessment scale—cognition
ADCS-ADL	Alzheimer’s disease co-operative study: activities of daily living
MDS-UPDRS	Movement disorders society-unified Parkinson’s disease rating scale
PBAs	Problem behaviors assessment-short
DDR1	Discoidin domain receptor-1
p-tau	Hyper-phosphorylated tau
IL	Interleukins
MMP	Matrix metalloproteases
DA	Dopamine
HVA	Homovanillic acid
AEs	Adverse Events
