# Supplementary material for: Safety, Cognitive, and Behavioral Outcomes in Patients with Dementia with Lewy Bodies Treated with Nilotinib
Source: J Clin Med. 2025 Jun 14;14(12):4245. doi: 10.3390/jcm14124245 (PMC12194573; doi:10.3390/jcm14124245)
Supplement: Supplementary file 1 [file jcm-14-04245-s001.zip › jcm-3683509-supplementary.pptx]

## Slide 1
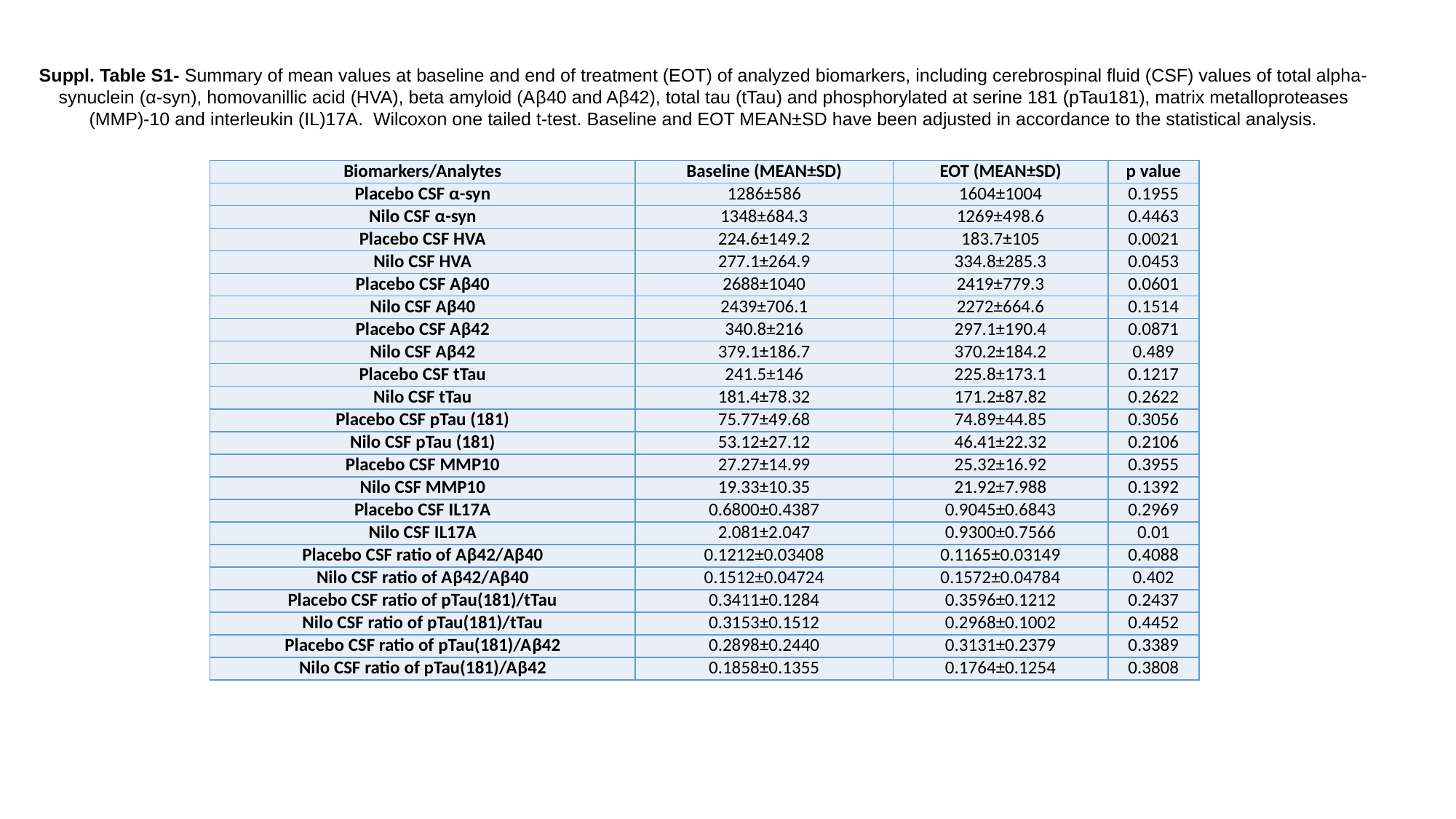

Suppl. Table S1- Summary of mean values at baseline and end of treatment (EOT) of analyzed biomarkers, including cerebrospinal fluid (CSF) values of total alpha-synuclein (α-syn), homovanillic acid (HVA), beta amyloid (Aβ40 and Aβ42), total tau (tTau) and phosphorylated at serine 181 (pTau181), matrix metalloproteases (MMP)-10 and interleukin (IL)17A. Wilcoxon one tailed t-test. Baseline and EOT MEAN±SD have been adjusted in accordance to the statistical analysis.
| Biomarkers/Analytes | Baseline (MEAN±SD) | EOT (MEAN±SD) | p value |
| --- | --- | --- | --- |
| Placebo CSF α-syn | 1286±586 | 1604±1004 | 0.1955 |
| Nilo CSF α-syn | 1348±684.3 | 1269±498.6 | 0.4463 |
| Placebo CSF HVA | 224.6±149.2 | 183.7±105 | 0.0021 |
| Nilo CSF HVA | 277.1±264.9 | 334.8±285.3 | 0.0453 |
| Placebo CSF Aβ40 | 2688±1040 | 2419±779.3 | 0.0601 |
| Nilo CSF Aβ40 | 2439±706.1 | 2272±664.6 | 0.1514 |
| Placebo CSF Aβ42 | 340.8±216 | 297.1±190.4 | 0.0871 |
| Nilo CSF Aβ42 | 379.1±186.7 | 370.2±184.2 | 0.489 |
| Placebo CSF tTau | 241.5±146 | 225.8±173.1 | 0.1217 |
| Nilo CSF tTau | 181.4±78.32 | 171.2±87.82 | 0.2622 |
| Placebo CSF pTau (181) | 75.77±49.68 | 74.89±44.85 | 0.3056 |
| Nilo CSF pTau (181) | 53.12±27.12 | 46.41±22.32 | 0.2106 |
| Placebo CSF MMP10 | 27.27±14.99 | 25.32±16.92 | 0.3955 |
| Nilo CSF MMP10 | 19.33±10.35 | 21.92±7.988 | 0.1392 |
| Placebo CSF IL17A | 0.6800±0.4387 | 0.9045±0.6843 | 0.2969 |
| Nilo CSF IL17A | 2.081±2.047 | 0.9300±0.7566 | 0.01 |
| Placebo CSF ratio of Aβ42/Aβ40 | 0.1212±0.03408 | 0.1165±0.03149 | 0.4088 |
| Nilo CSF ratio of Aβ42/Aβ40 | 0.1512±0.04724 | 0.1572±0.04784 | 0.402 |
| Placebo CSF ratio of pTau(181)/tTau | 0.3411±0.1284 | 0.3596±0.1212 | 0.2437 |
| Nilo CSF ratio of pTau(181)/tTau | 0.3153±0.1512 | 0.2968±0.1002 | 0.4452 |
| Placebo CSF ratio of pTau(181)/Aβ42 | 0.2898±0.2440 | 0.3131±0.2379 | 0.3389 |
| Nilo CSF ratio of pTau(181)/Aβ42 | 0.1858±0.1355 | 0.1764±0.1254 | 0.3808 |

## Slide 2
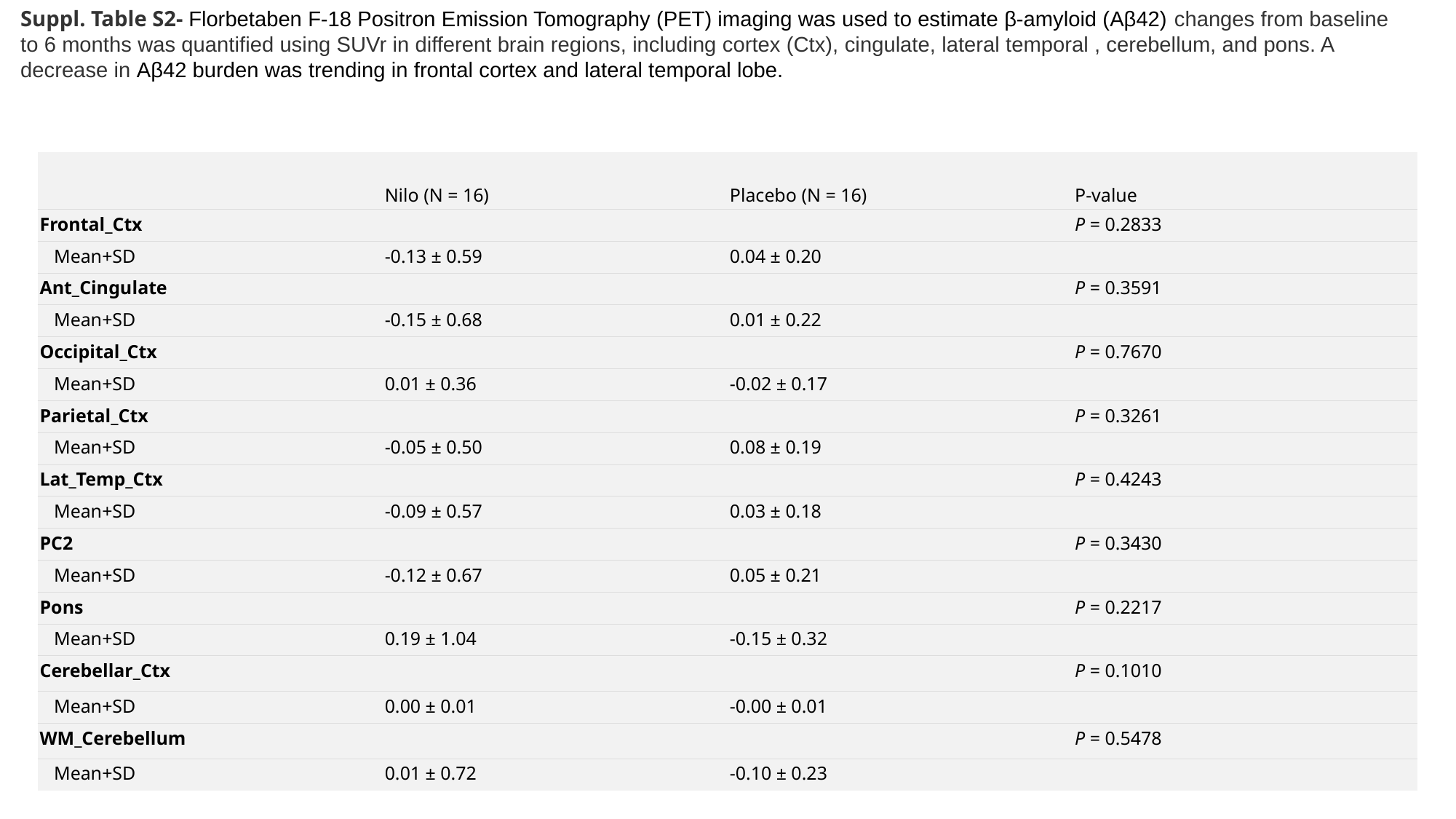

Suppl. Table S2- Florbetaben F-18 Positron Emission Tomography (PET) imaging was used to estimate β-amyloid (Aβ42) changes from baseline to 6 months was quantified using SUVr in different brain regions, including cortex (Ctx), cingulate, lateral temporal , cerebellum, and pons. A decrease in Aβ42 burden was trending in frontal cortex and lateral temporal lobe.
| | Nilo (N = 16) | Placebo (N = 16) | P-value |
| --- | --- | --- | --- |
| Frontal\_Ctx | | | P = 0.2833 |
| Mean+SD | -0.13 ± 0.59 | 0.04 ± 0.20 | |
| Ant\_Cingulate | | | P = 0.3591 |
| Mean+SD | -0.15 ± 0.68 | 0.01 ± 0.22 | |
| Occipital\_Ctx | | | P = 0.7670 |
| Mean+SD | 0.01 ± 0.36 | -0.02 ± 0.17 | |
| Parietal\_Ctx | | | P = 0.3261 |
| Mean+SD | -0.05 ± 0.50 | 0.08 ± 0.19 | |
| Lat\_Temp\_Ctx | | | P = 0.4243 |
| Mean+SD | -0.09 ± 0.57 | 0.03 ± 0.18 | |
| PC2 | | | P = 0.3430 |
| Mean+SD | -0.12 ± 0.67 | 0.05 ± 0.21 | |
| Pons | | | P = 0.2217 |
| Mean+SD | 0.19 ± 1.04 | -0.15 ± 0.32 | |
| Cerebellar\_Ctx | | | P = 0.1010 |
| Mean+SD | 0.00 ± 0.01 | -0.00 ± 0.01 | |
| WM\_Cerebellum | | | P = 0.5478 |
| Mean+SD | 0.01 ± 0.72 | -0.10 ± 0.23 | |

## Slide 3
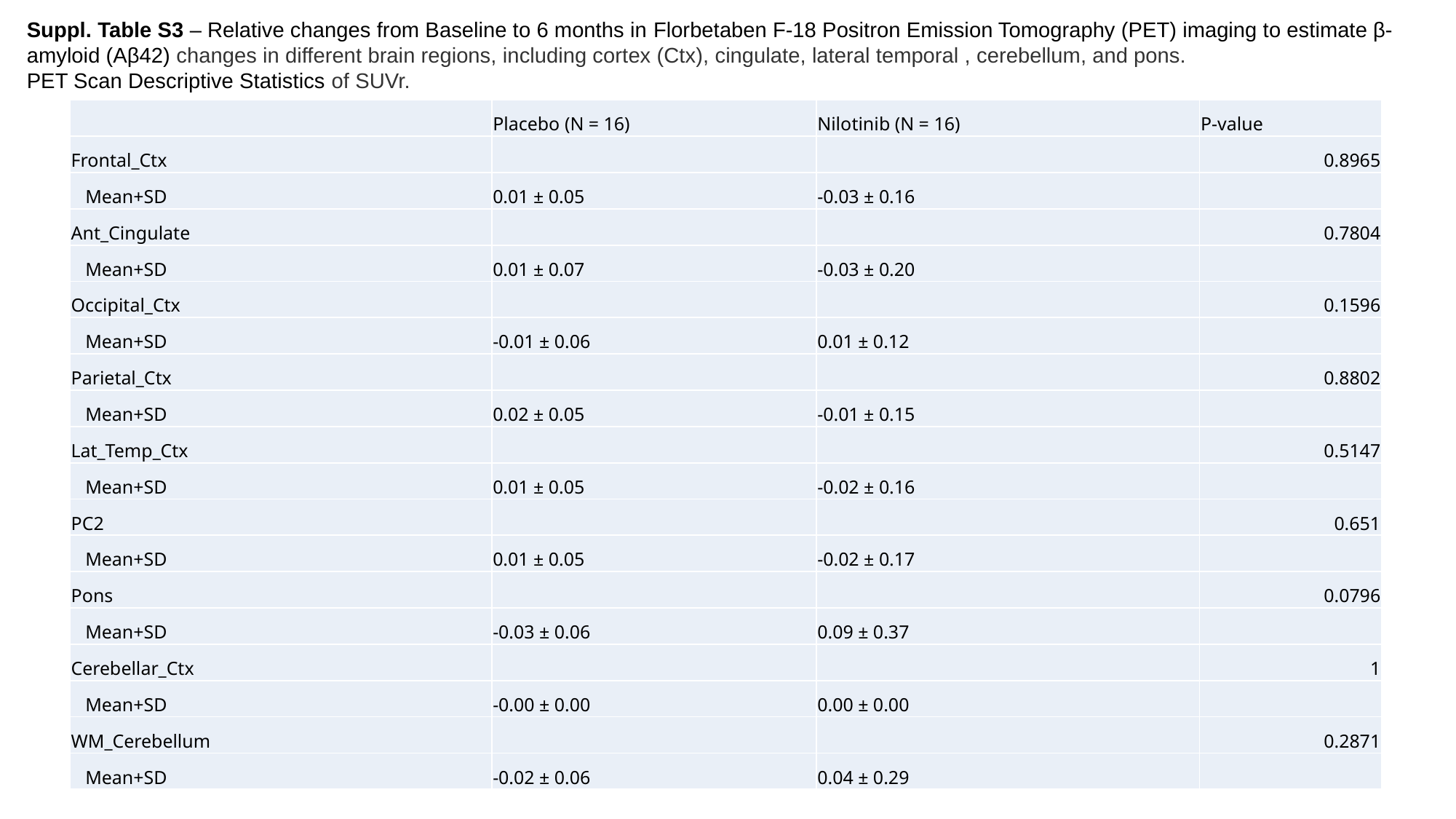

Suppl. Table S3 – Relative changes from Baseline to 6 months in Florbetaben F-18 Positron Emission Tomography (PET) imaging to estimate β-amyloid (Aβ42) changes in different brain regions, including cortex (Ctx), cingulate, lateral temporal , cerebellum, and pons.
PET Scan Descriptive Statistics of SUVr.
| | Placebo (N = 16) | Nilotinib (N = 16) | P-value |
| --- | --- | --- | --- |
| Frontal\_Ctx | | | 0.8965 |
| Mean+SD | 0.01 ± 0.05 | -0.03 ± 0.16 | |
| Ant\_Cingulate | | | 0.7804 |
| Mean+SD | 0.01 ± 0.07 | -0.03 ± 0.20 | |
| Occipital\_Ctx | | | 0.1596 |
| Mean+SD | -0.01 ± 0.06 | 0.01 ± 0.12 | |
| Parietal\_Ctx | | | 0.8802 |
| Mean+SD | 0.02 ± 0.05 | -0.01 ± 0.15 | |
| Lat\_Temp\_Ctx | | | 0.5147 |
| Mean+SD | 0.01 ± 0.05 | -0.02 ± 0.16 | |
| PC2 | | | 0.651 |
| Mean+SD | 0.01 ± 0.05 | -0.02 ± 0.17 | |
| Pons | | | 0.0796 |
| Mean+SD | -0.03 ± 0.06 | 0.09 ± 0.37 | |
| Cerebellar\_Ctx | | | 1 |
| Mean+SD | -0.00 ± 0.00 | 0.00 ± 0.00 | |
| WM\_Cerebellum | | | 0.2871 |
| Mean+SD | -0.02 ± 0.06 | 0.04 ± 0.29 | |

## Slide 4
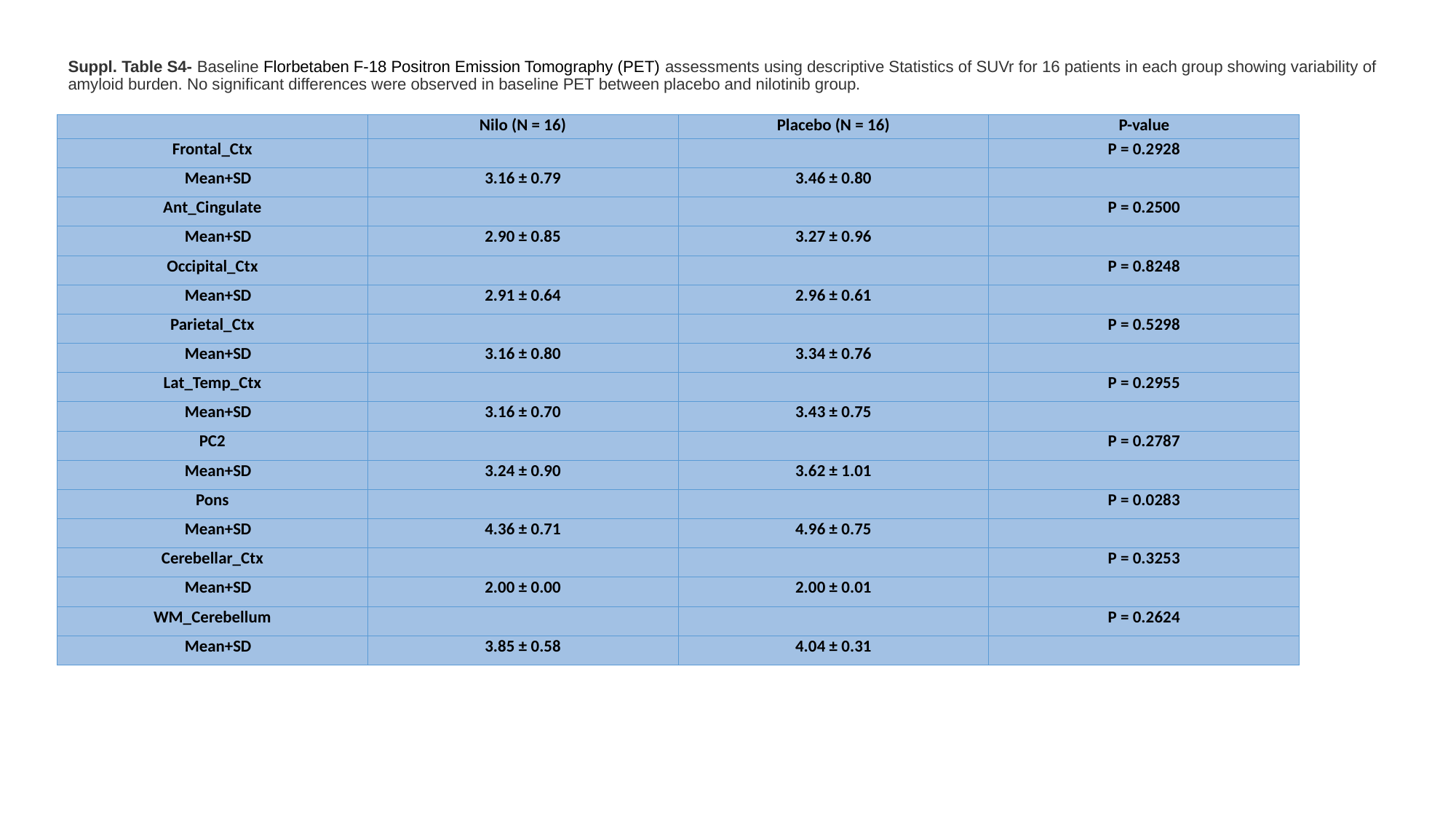

# Suppl. Table S4- Baseline Florbetaben F-18 Positron Emission Tomography (PET) assessments using descriptive Statistics of SUVr for 16 patients in each group showing variability of amyloid burden. No significant differences were observed in baseline PET between placebo and nilotinib group.
| | Nilo (N = 16) | Placebo (N = 16) | P-value |
| --- | --- | --- | --- |
| Frontal\_Ctx | | | P = 0.2928 |
| Mean+SD | 3.16 ± 0.79 | 3.46 ± 0.80 | |
| Ant\_Cingulate | | | P = 0.2500 |
| Mean+SD | 2.90 ± 0.85 | 3.27 ± 0.96 | |
| Occipital\_Ctx | | | P = 0.8248 |
| Mean+SD | 2.91 ± 0.64 | 2.96 ± 0.61 | |
| Parietal\_Ctx | | | P = 0.5298 |
| Mean+SD | 3.16 ± 0.80 | 3.34 ± 0.76 | |
| Lat\_Temp\_Ctx | | | P = 0.2955 |
| Mean+SD | 3.16 ± 0.70 | 3.43 ± 0.75 | |
| PC2 | | | P = 0.2787 |
| Mean+SD | 3.24 ± 0.90 | 3.62 ± 1.01 | |
| Pons | | | P = 0.0283 |
| Mean+SD | 4.36 ± 0.71 | 4.96 ± 0.75 | |
| Cerebellar\_Ctx | | | P = 0.3253 |
| Mean+SD | 2.00 ± 0.00 | 2.00 ± 0.01 | |
| WM\_Cerebellum | | | P = 0.2624 |
| Mean+SD | 3.85 ± 0.58 | 4.04 ± 0.31 | |

## Slide 5
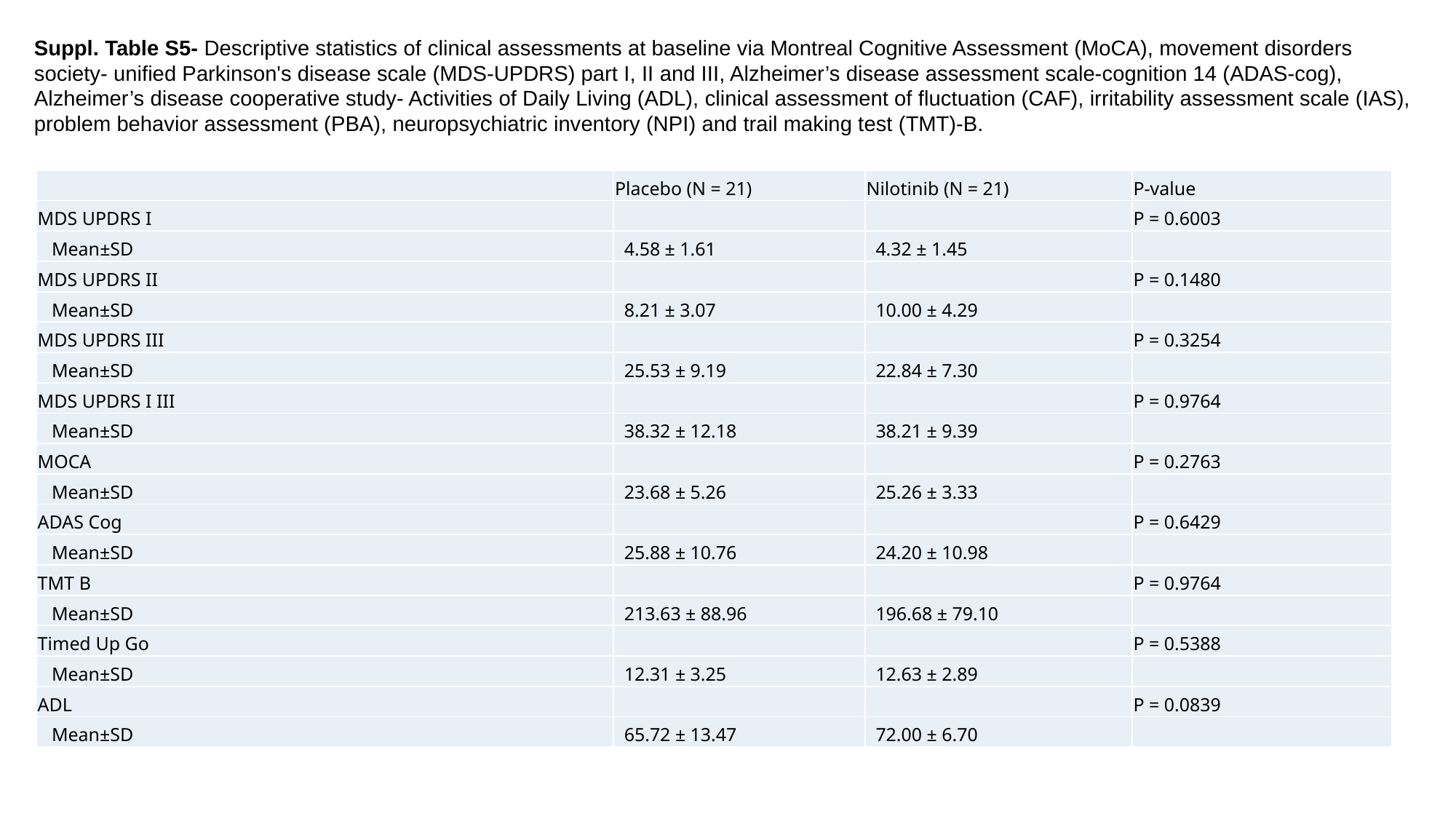

Suppl. Table S5- Descriptive statistics of clinical assessments at baseline via Montreal Cognitive Assessment (MoCA), movement disorders society- unified Parkinson's disease scale (MDS-UPDRS) part I, II and III, Alzheimer’s disease assessment scale-cognition 14 (ADAS-cog), Alzheimer’s disease cooperative study- Activities of Daily Living (ADL), clinical assessment of fluctuation (CAF), irritability assessment scale (IAS), problem behavior assessment (PBA), neuropsychiatric inventory (NPI) and trail making test (TMT)-B.
| | Placebo (N = 21) | Nilotinib (N = 21) | P-value |
| --- | --- | --- | --- |
| MDS UPDRS I | | | P = 0.6003 |
| Mean±SD | 4.58 ± 1.61 | 4.32 ± 1.45 | |
| MDS UPDRS II | | | P = 0.1480 |
| Mean±SD | 8.21 ± 3.07 | 10.00 ± 4.29 | |
| MDS UPDRS III | | | P = 0.3254 |
| Mean±SD | 25.53 ± 9.19 | 22.84 ± 7.30 | |
| MDS UPDRS I III | | | P = 0.9764 |
| Mean±SD | 38.32 ± 12.18 | 38.21 ± 9.39 | |
| MOCA | | | P = 0.2763 |
| Mean±SD | 23.68 ± 5.26 | 25.26 ± 3.33 | |
| ADAS Cog | | | P = 0.6429 |
| Mean±SD | 25.88 ± 10.76 | 24.20 ± 10.98 | |
| TMT B | | | P = 0.9764 |
| Mean±SD | 213.63 ± 88.96 | 196.68 ± 79.10 | |
| Timed Up Go | | | P = 0.5388 |
| Mean±SD | 12.31 ± 3.25 | 12.63 ± 2.89 | |
| ADL | | | P = 0.0839 |
| Mean±SD | 65.72 ± 13.47 | 72.00 ± 6.70 | |

## Slide 6
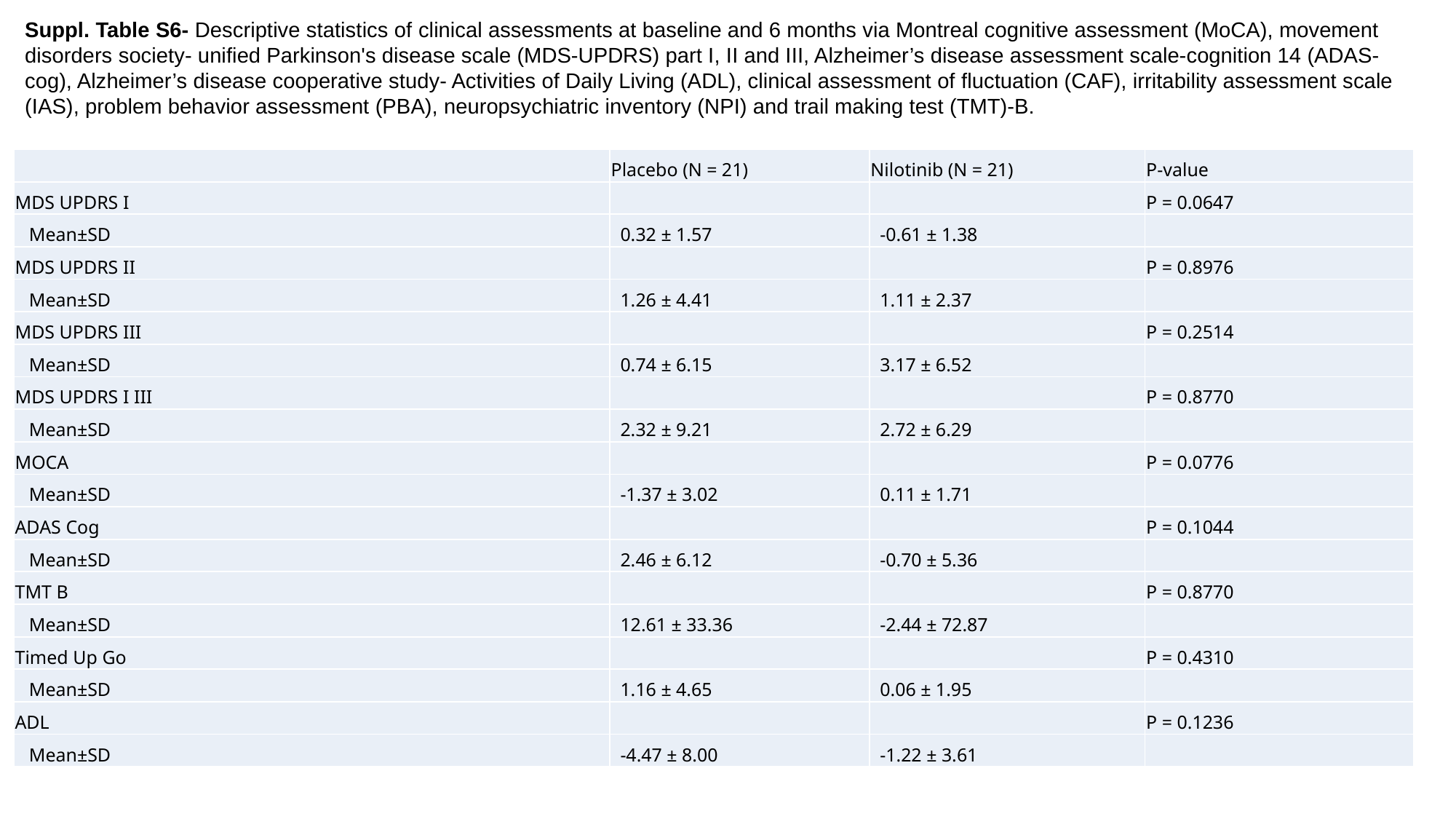

Suppl. Table S6- Descriptive statistics of clinical assessments at baseline and 6 months via Montreal cognitive assessment (MoCA), movement disorders society- unified Parkinson's disease scale (MDS-UPDRS) part I, II and III, Alzheimer’s disease assessment scale-cognition 14 (ADAS-cog), Alzheimer’s disease cooperative study- Activities of Daily Living (ADL), clinical assessment of fluctuation (CAF), irritability assessment scale (IAS), problem behavior assessment (PBA), neuropsychiatric inventory (NPI) and trail making test (TMT)-B.
| | Placebo (N = 21) | Nilotinib (N = 21) | P-value |
| --- | --- | --- | --- |
| MDS UPDRS I | | | P = 0.0647 |
| Mean±SD | 0.32 ± 1.57 | -0.61 ± 1.38 | |
| MDS UPDRS II | | | P = 0.8976 |
| Mean±SD | 1.26 ± 4.41 | 1.11 ± 2.37 | |
| MDS UPDRS III | | | P = 0.2514 |
| Mean±SD | 0.74 ± 6.15 | 3.17 ± 6.52 | |
| MDS UPDRS I III | | | P = 0.8770 |
| Mean±SD | 2.32 ± 9.21 | 2.72 ± 6.29 | |
| MOCA | | | P = 0.0776 |
| Mean±SD | -1.37 ± 3.02 | 0.11 ± 1.71 | |
| ADAS Cog | | | P = 0.1044 |
| Mean±SD | 2.46 ± 6.12 | -0.70 ± 5.36 | |
| TMT B | | | P = 0.8770 |
| Mean±SD | 12.61 ± 33.36 | -2.44 ± 72.87 | |
| Timed Up Go | | | P = 0.4310 |
| Mean±SD | 1.16 ± 4.65 | 0.06 ± 1.95 | |
| ADL | | | P = 0.1236 |
| Mean±SD | -4.47 ± 8.00 | -1.22 ± 3.61 | |

## Slide 7
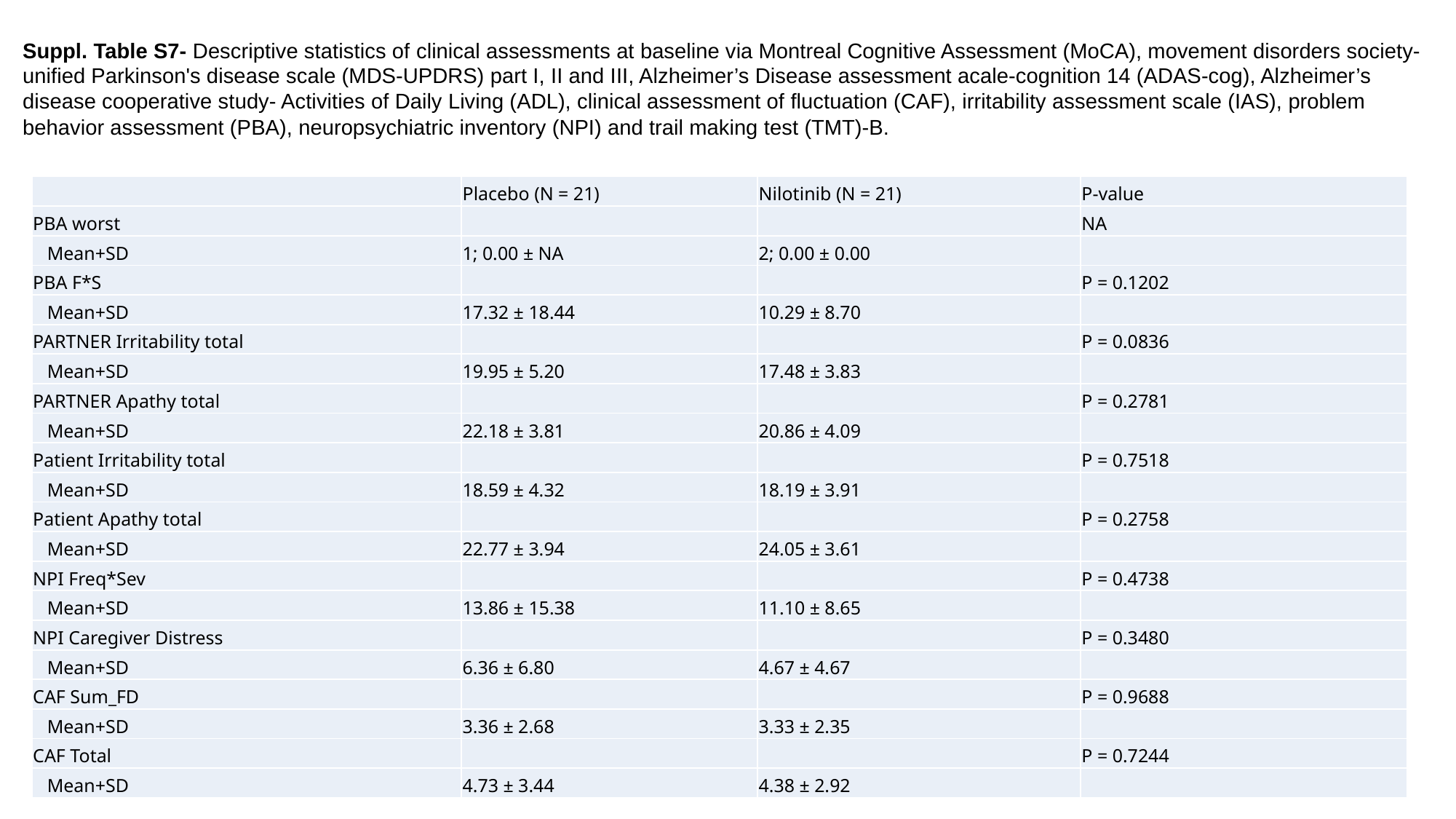

Suppl. Table S7- Descriptive statistics of clinical assessments at baseline via Montreal Cognitive Assessment (MoCA), movement disorders society- unified Parkinson's disease scale (MDS-UPDRS) part I, II and III, Alzheimer’s Disease assessment acale-cognition 14 (ADAS-cog), Alzheimer’s disease cooperative study- Activities of Daily Living (ADL), clinical assessment of fluctuation (CAF), irritability assessment scale (IAS), problem behavior assessment (PBA), neuropsychiatric inventory (NPI) and trail making test (TMT)-B.
| | Placebo (N = 21) | Nilotinib (N = 21) | P-value |
| --- | --- | --- | --- |
| PBA worst | | | NA |
| Mean+SD | 1; 0.00 ± NA | 2; 0.00 ± 0.00 | |
| PBA F\*S | | | P = 0.1202 |
| Mean+SD | 17.32 ± 18.44 | 10.29 ± 8.70 | |
| PARTNER Irritability total | | | P = 0.0836 |
| Mean+SD | 19.95 ± 5.20 | 17.48 ± 3.83 | |
| PARTNER Apathy total | | | P = 0.2781 |
| Mean+SD | 22.18 ± 3.81 | 20.86 ± 4.09 | |
| Patient Irritability total | | | P = 0.7518 |
| Mean+SD | 18.59 ± 4.32 | 18.19 ± 3.91 | |
| Patient Apathy total | | | P = 0.2758 |
| Mean+SD | 22.77 ± 3.94 | 24.05 ± 3.61 | |
| NPI Freq\*Sev | | | P = 0.4738 |
| Mean+SD | 13.86 ± 15.38 | 11.10 ± 8.65 | |
| NPI Caregiver Distress | | | P = 0.3480 |
| Mean+SD | 6.36 ± 6.80 | 4.67 ± 4.67 | |
| CAF Sum\_FD | | | P = 0.9688 |
| Mean+SD | 3.36 ± 2.68 | 3.33 ± 2.35 | |
| CAF Total | | | P = 0.7244 |
| Mean+SD | 4.73 ± 3.44 | 4.38 ± 2.92 | |

## Slide 8
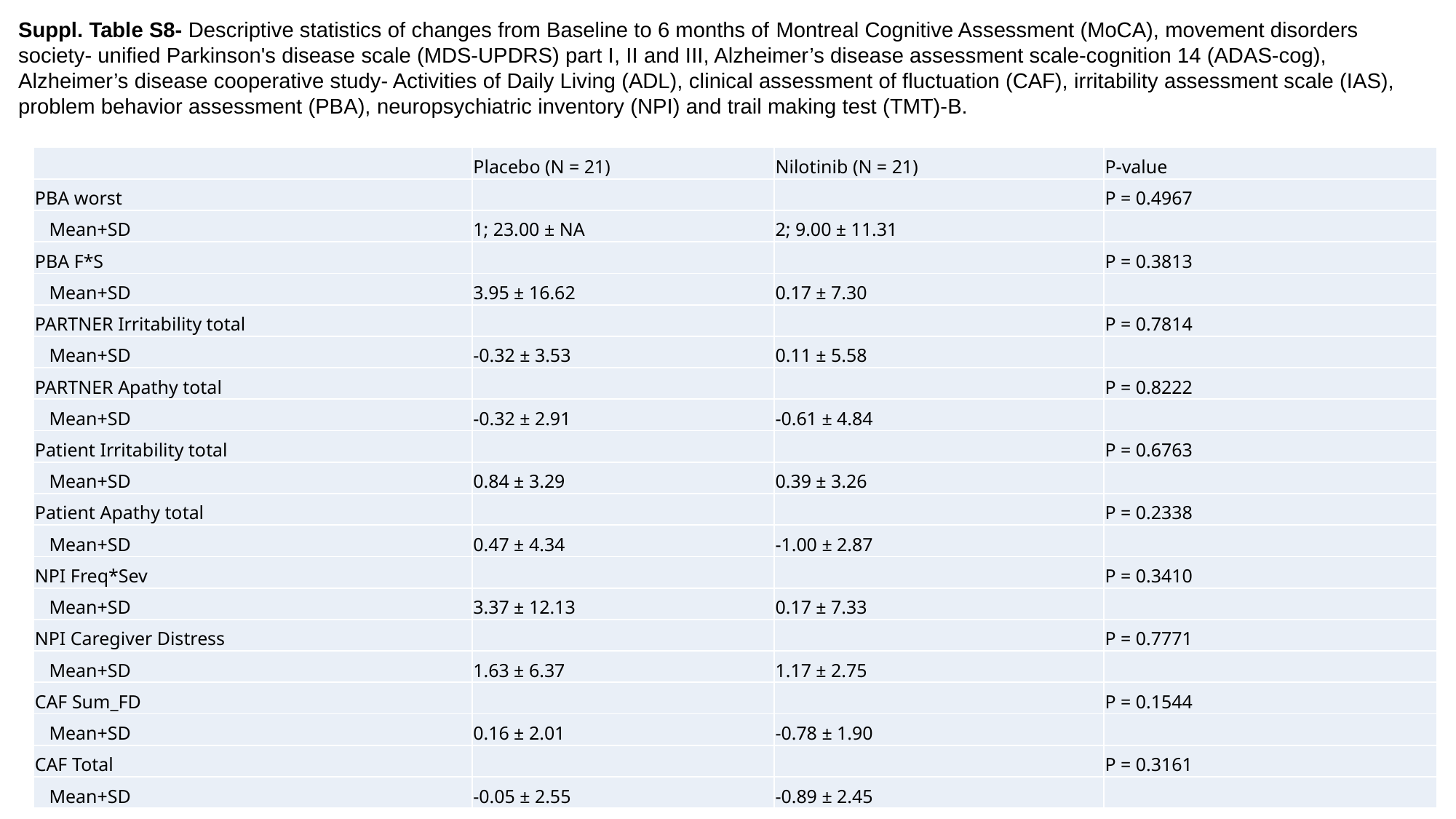

Suppl. Table S8- Descriptive statistics of changes from Baseline to 6 months of Montreal Cognitive Assessment (MoCA), movement disorders society- unified Parkinson's disease scale (MDS-UPDRS) part I, II and III, Alzheimer’s disease assessment scale-cognition 14 (ADAS-cog), Alzheimer’s disease cooperative study- Activities of Daily Living (ADL), clinical assessment of fluctuation (CAF), irritability assessment scale (IAS), problem behavior assessment (PBA), neuropsychiatric inventory (NPI) and trail making test (TMT)-B.
| | Placebo (N = 21) | Nilotinib (N = 21) | P-value |
| --- | --- | --- | --- |
| PBA worst | | | P = 0.4967 |
| Mean+SD | 1; 23.00 ± NA | 2; 9.00 ± 11.31 | |
| PBA F\*S | | | P = 0.3813 |
| Mean+SD | 3.95 ± 16.62 | 0.17 ± 7.30 | |
| PARTNER Irritability total | | | P = 0.7814 |
| Mean+SD | -0.32 ± 3.53 | 0.11 ± 5.58 | |
| PARTNER Apathy total | | | P = 0.8222 |
| Mean+SD | -0.32 ± 2.91 | -0.61 ± 4.84 | |
| Patient Irritability total | | | P = 0.6763 |
| Mean+SD | 0.84 ± 3.29 | 0.39 ± 3.26 | |
| Patient Apathy total | | | P = 0.2338 |
| Mean+SD | 0.47 ± 4.34 | -1.00 ± 2.87 | |
| NPI Freq\*Sev | | | P = 0.3410 |
| Mean+SD | 3.37 ± 12.13 | 0.17 ± 7.33 | |
| NPI Caregiver Distress | | | P = 0.7771 |
| Mean+SD | 1.63 ± 6.37 | 1.17 ± 2.75 | |
| CAF Sum\_FD | | | P = 0.1544 |
| Mean+SD | 0.16 ± 2.01 | -0.78 ± 1.90 | |
| CAF Total | | | P = 0.3161 |
| Mean+SD | -0.05 ± 2.55 | -0.89 ± 2.45 | |
